# Repurposing development genes for axonal regeneration following injury: Examining the roles of Wnt signaling

**DOI:** 10.3389/fcell.2024.1417928

**Published:** 2024-05-31

**Authors:** Gabrielle A. Albano, Abigail S. Hackam

**Affiliations:** Bascom Palmer Eye Institute, University of Miami Miller School of Medicine, Miami, FL, United States

**Keywords:** axon regeneration, embryonic development, Wnt signaling, Müller glia, retina, spinal cord, brain

## Abstract

In this review, we explore the connections between developmental embryology and axonal regeneration. Genes that regulate embryogenesis and central nervous system (CNS) development are discussed for their therapeutic potential to induce axonal and cellular regeneration in adult tissues after neuronal injury. Despite substantial differences in the tissue environment in the developing CNS compared with the injured CNS, recent studies have identified multiple molecular pathways that promote axonal growth in both scenarios. We describe various molecular cues and signaling pathways involved in neural development, with an emphasis on the versatile Wnt signaling pathway. We discuss the capacity of developmental factors to initiate axonal regrowth in adult neural tissue within the challenging environment of the injured CNS. Our discussion explores the roles of Wnt signaling and also examines the potential of other embryonic genes including Pax, BMP, Ephrin, SOX, CNTF, PTEN, mTOR and STAT3 to contribute to axonal regeneration in various CNS injury model systems, including spinal cord and optic crush injuries in mice, *Xenopus* and zebrafish. Additionally, we describe potential contributions of Müller glia redifferentiation to neuronal regeneration after injury. Therefore, this review provides a comprehensive summary of the state of the field, and highlights promising research directions for the potential therapeutic applications of specific embryologic molecular pathways in axonal regeneration in adults.

## Introduction to embryonic development

The application of developmental embryology to the field of axonal regeneration has the potential to uncover new insights into molecular pathways that may have the capacity to induce axon injury repair and regrowth. We begin with an overview of molecular signaling pathways involved in embryonic and CNS development then describe evidence for repurposing specific molecular factors for inducing axonal and cellular regeneration after neuronal injury.

Embryonic development requires numerous precisely coordinated physiological and molecular signals. The first stage of vertebrate development after fertilization is the formation of a zygote that undergoes multiple rounds of cell cleavage to form a blastula, which further differentiates to create cavities and a bilaminar disc (Gilbert, 2024). The process of gastrulation begins when the bilaminar disc involutes along the primitive streak creating a trilaminar germ layer disc. The trilaminar disc then gives rise to the ectoderm, mesoderm and endodermal layers. Further differentiation of these layers is the basis for development of all organs in the body. The notochord arises from the axial mesoderm and begins the process of forming the spinal cord. The notochord induces the ectodermal layer to thicken and form the neural folds that lead to the neural tube, which gives rise to the spinal cord and brain. During neural fold closure, neural crest cells emerge and differentiate into various cell types, including melanocytes, craniofacial bone and cartilage, glia, and neurons (Ramesh et al., 2017). Multiple ligands, cellular signaling pathways and transcription factors are essential for precisely coordinating each step of embryonic development. One of the many signaling pathways that mediate development is the Wnt signaling pathway.

### Wnt signaling pathways

Wnt ligands contribute to embryonic patterning, axonal growth, cellular determination, differentiation and proliferation. Of the various developmental and regenerative molecules studied, there is more research reported in the literature on the roles of Wnt pathways in both embryogenesis and axonal regeneration. Wnt signaling is upstream of many developmental and axon regenerative genes, although there is no evidence that Wnt signaling has a more central role than other signaling pathways. The next section focuses on the roles of Wnt signaling during development to illustrate potential applications of repurposing developmental genes for axonal regrowth after injury in adults.

**FIGURE 1:**
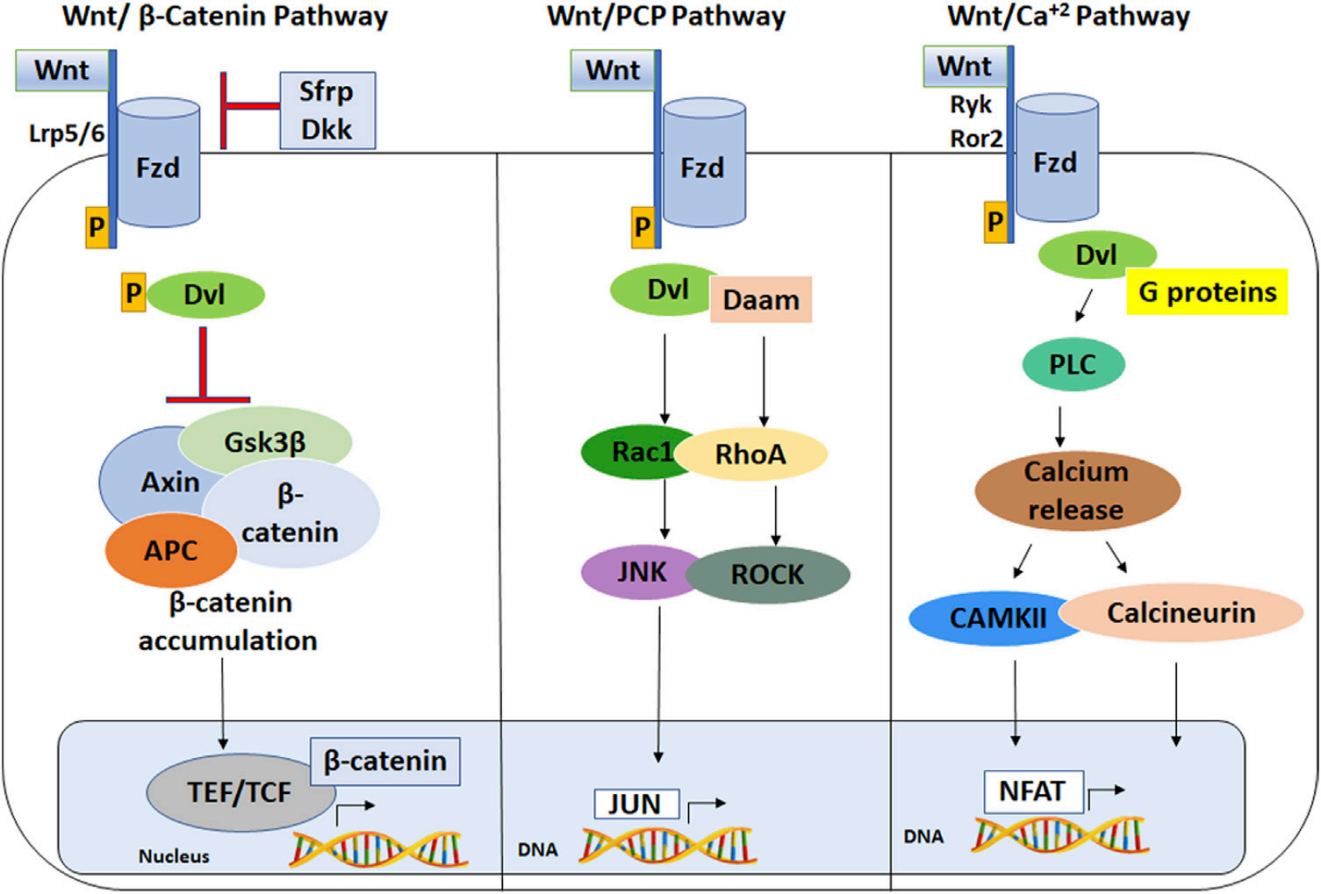
Wnt signaling pathways. (Left) The Wnt/β-Catenin Pathway. Wnt ligand binds at the plasma membrane to Lrp5/6 and Fzd co-receptors, which phosphorylates and activates Dishevelled (Dvl). Activated Dvl inhibits APC, GSKβ, and the axin complex resulting in accumulation of stable β-catenin, which translocates into the nucleus to interact with Tcf/Lef family of transcription factors, leading to transcriptional activation of target genes. (Middle) The Wnt/planar cell polarity pathway is stimulated by a Wnt ligand binding to Fzd receptor, which activates Dvl and Daam, leading to Jun-mediated gene expression. (Right) The Wnt/calcium pathway stimulates calcium release from endoplasmic reticulum and leads to NFAT-mediated gene expression. LRP5/6: lipoprotein receptor related protein five or 6; Fzd: Frizzled; Dvl: Dishevelled; APC: adenomatous polyposis coli; Gsk3: glycogen synthase kinase 3; Ror2: receptor tyrosine kinase-like orphan receptor 2; NFAT: nuclear factor of activated T-cells; CaMKII: CaM kinase II; RhoA: Ras homolog gene family, member A.

Wnt ligands secreted by various cell types bind to Frizzled (Fzd) receptors on the plasma membrane of their target cells, which leads to activation of specific signaling cascades and induction of target gene expression (MacDonald et al., 2009). The best characterized Wnt signaling pathway is the canonical Wnt/β-catenin pathway, in which binding of one of over a dozen different types of Wnt ligands to one of seven types of Fzd receptors and a LRP5/6 coreceptor results in phosphorylation of the Dishevelled (Dvl) adapter protein (Figure 1). Dvl associates with regulatory proteins that result in inhibition of glycogen synthase kinase-3β (GSK3β), which leads to stabilization and nuclear transport of β-catenin. After entering the nucleus, β-catenin interacts with TCF/LEF transcription factor family members to regulate expression of specific Wnt target genes (Patapoutian and Reichardt, 2000). Wnt target genes include transcription factors and cell cycling regulators that mediate processes such as cell growth, cell proliferation and differentiation (Patapoutian and Reichardt, 2000).

**FIGURE 2:**
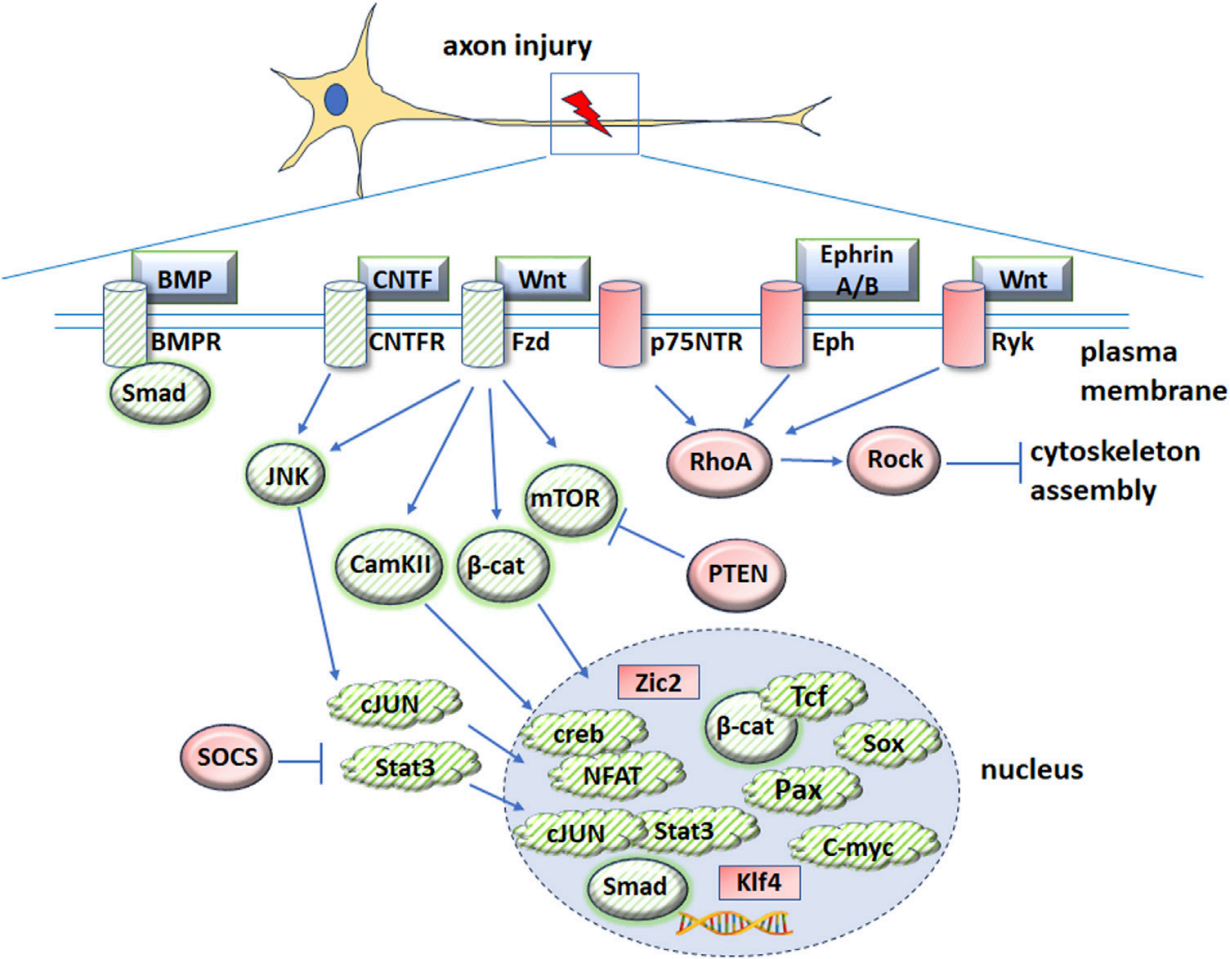
Schematic diagram illustrating genes and signaling pathways implicated in both embryogenesis and axonal regeneration. Proteins that promote axonal regeneration are indicated by green cross-hatches and proteins that are inhibitory are indicated by red shading. Downstream intermediators and transcription factors are indicated by blue arrows. To enhance clarity, most signaling pathway inhibitors and regulators are not included. Please see the text for further information and abbreviations.

Wnt ligands also act through several non-canonical pathways, including the Wnt/planar cell polarity (PCP) and Wnt/Ca^2+^ pathways (Figure 1) (Akoumianakis et al., 2022). The Wnt/PCP pathway is induced by Wnt ligands interacting with specific Fzd receptors and a co-receptor such as ROR1/2 or Ryk, which leads to multiple downstream reactions including activation of Rho and Rac GTPases, Rho-Kinase (ROCK) and c-Jun N-terminal kinase (JNK). The PCP pathway is implicated in developmental processes such as vertebrate gastrulation and *Drosophila* ommatidia development (Jenny, 2010; Navajas et al., 2019). The Wnt-PCP pathway is also involved in regulating neuronal polarity in developing *C. elegans*, synaptogenesis and axonal growth cone guidance in mammals and flies (He et al., 2018). By comparison, the Wnt/Ca^2+^ pathway is induced by Wnt-Fzd binding and activation of phospholipase C (PLC), which stimulates secondary messenger proteins that cause calcium influx into the cytoplasm through intracellular stores. Increased calcium induces activation of PKC and Ca^2+^/calmodulin-dependent protein kinase II (CaMKII) and calcium-dependent transcription factors such as NFKB1, CREB and NFAT (Hackam, 2005). The non-canonical Wnt/Ca^2+^ pathway has been implicated in embryonic processes such as neuronal cell migration and fate, axon and dendritic growth, axonal guidance and synapse formation (Rosso and Inestrosa, 2013; Garcia et al., 2018). The roles of Wnt ligands in neural development depend on whether canonical or non-canonical pathways are stimulated (Figure 2), which is influenced by receptor expression level, Wnt ligand dose and co-expression of different Wnt ligands (Patapoutian and Reichardt, 2000; Holmen et al., 2002; Kestler and Kühl, 2011; Niehrs, 2012).

### Early embryonic neural development

The first step of axial patterning begins with activation in early development and has been best described in *Xenopus*. The Nieuwkoop center in *Xenopus* embryos induces a group of dorsal vegetal cells called an organizer that initiates neural tissue development and establishes dorso-ventral polarity of the embryo (Tam and Behringer, 1997). The equivalent function of the *Xenopus* Nieuwkoop center in chicks is performed by the marginal zone cells. The chick Vg1 homolog is expressed within the posterior marginal zone cells and in the adjacent Koller’s sickle. Posterior marginal zone cells may induce formation of the primitive streak via Vg1 (Tam and Behringer, 1997).

Wnt signaling is one of several pathways that are crucial to dorsal-ventral axial patterning during embryogenesis. Wnt signaling is present during primitive streak formation in cultured embryoid bodies from embryonic stem cells and functions to control epithelial to mesenchymal transition, which is necessary for gastrulation and axial patterning (ten Berge et al., 2008). Various studies examined how Wnt signaling influence on gastrulation leads to axial patterning. For example, ectopic administration of Wnt8 ligand induced an entirely distinct secondary embryonic axis in *Xenopus*, highlighting the role of Wnt signaling in axial patterning and head and trunk development (Sokol et al., 1991).

Studies in developing *Xenopus* and mice investigated the role of Wnt signaling timing and concentration of Wnt ligands on axial patterning. Wnt8 injection in *Xenopus* embryos at early stages of cell development resulted in dorsalization in organizer cells, but injection of Wnt8 at late stages resulted in an antagonistic effect on the organizer. Delivery of Wnt8 at later stages, following the midblastula, led to ventralization of dorsal mesodermal cells instead of dorsalization as seen in earlier stages (Kiecker and Niehrs, 2001). This result demonstrated that cells fated to become notochord were instead induced ventrally to be muscle, resulting in embryos without heads and notochords. Removal of Wnt8 resulted in anteriorization of embryos, leading to smaller somatic muscles and enlarged heads, indicating that Wnt signaling is responsible for somatic muscle formation (Hoppler et al., 1996). Additionally, overexpression of Wnt8 in mice resulted in the absence of the anterior forebrain (Christian and Moon, 1993), indicating that both timing of Wnt signaling and level of ligand expression are important in axial patterning.

Wnt signaling plays a role in anterior-posterior axis formation in addition to dorsal-ventral axial patterning. Wnt ligands are expressed in the paraxial dorsolateral mesoderm underlying the neural plate in *Xenopus* embryos and Wnt signaling activation results in transcription of the Meis3 gene that is expressed in the neuroectoderm above. Meis3 activation permits posterization of cells creating an anterior-posterior axis (Elkouby et al., 2010). Wnt ligands show an expression gradient along the neural plate with high expression in the posterior axis and lower expression anteriorly. Significant impacts to neural development result from disruption or alteration of Wnt ligand expression levels. For example, the importance of Wnt ligand gradient in neuronal differentiation was demonstrated by injecting increasing doses of Wnt8 into *Xenopus* embryos, which resulted in different neural fates with increasing dosage (Kiecker and Niehrs, 2001). At low amounts of Wnt8, the anterior neural marker Bf1 was repressed and the mid-hindbrain marker En2 was induced, whereas at higher Wnt8 amounts, the anterior neural markers were repressed and midbrain marker Krox2 was expressed. These results demonstrate optimal doses of Wnt signaling are necessary for anterior-posterior neural patterning during gastrulation in *Xenopus* (Kiecker and Niehrs, 2001).

Numerous interactions among Wnt signaling and other key developmental signaling pathways during embryogenesis have been described. For example, Wnt signaling regulates other mediators of development such as Notch, JNK, BMPs, Ephrins and TGFβ (Garcia et al., 2018). An example of signaling pathway cross-talk during early development occurs during the closure of the neural tube. In this process, Ephrin B2 interacts with Dishevelled 2 (DVL2), which initiates the formation of a complex containing Wnt4, EphrinB2, Ror2, DVL2 and Shroom3 that facilitates neural tube closure. Reduced Wnt4 expression leads to significant neural tube defects, which can be rescued by Wnt4 injection (Yoon et al., 2023), indicating the importance of Wnt signaling in this process.

### Ocular development

Ocular development begins from the anterior neural plate during late gastrulation. Eye development commences with the formation of the visual field that emerges from the forebrain. Multiple transcription factors, including Rx, Pax6, Six3 and Optx2, coordinate with inhibitors and activators of BMP, Nodal and Wnt/β-catenin signaling to form a single field of eye development, and their roles have been described in several comprehensive reviews (Grogg et al., 2005; Rosso and Inestrosa, 2013). There are several lines of evidence that implicate Wnt signaling in ocular development. For example, inhibition of Wnt1 by direct interaction with Six3 in the anterior neural plate prevents posteriorization, and genetic loss of Six3 in mice results in abnormalities in forebrain formation, loss of eyes and nose and reduced eye field development (Lagutin et al., 2003; Fuhrmann, 2008). Similarly, elimination of the Wnt/β-catenin transcriptional activator Pygopus in *Xenopus* embryos led to brain defects, including lack of eyes, providing further evidence that Wnt signaling is necessary for eye development (Lake and Kao, 2003).

Retina formation occurs by invagination of the eye primordia, which forms two optic vesicles that further invaginate after interacting with the overlaying surface ectoderm. This tissue reorganization results in two layers: the inner layer, which ultimately becomes the retina, and the outer layer, which becomes the non-neural retinal pigmented epithelium (Fuhrmann, 2008). Analysis of various animal models demonstrated an array of different signaling pathways involved in retinal development. Notably, Wnt signaling is a common active pathway driving retinal formation and maturation. In chicken and mice, the canonical Wnt pathway activates TCF/LEF family members in the dorsal optic vesicle, which will later become the optic cup (Cho and Cepko, 2006). In mice, ablation of the Wnt/β-catenin coreceptor LRP6 causes loss of TCF/LEF expression in the dorsal optic vesicle, which inhibits optic cup formation (Pinson et al., 2000). Additional studies demonstrated that loss of LRP6 in mice resulted in microphthalmia and other developmental defects of the limbs and axial skeleton (Pinson et al., 2000). Wnt signaling is also necessary for the formation of the optic cup in zebrafish. Inhibition of Wnt signaling by targeting porcupine, a factor required for Wnt ligand secretion, leads to deficiencies in optic fissure formation and defects in the optic cup (Eckert et al., 2019). These findings indicate that Wnt signaling plays an important role in retinal development and its function is conserved across species.

### Embryonic axonal growth

The process of axonogenesis in the developing nervous system can be divided into several distinct phases: i) Initiation of axon sprouting from the polarized soma of the neuroblast, often occurring prior to neuroblast migration is complete. Multiple pathways are involved in axon sprouting that regulate cytoskeletal dynamics and gene transcription, including PI3K signaling, JAK/STAT, MAPK/ERK, semaphorin-plexin, Ephrins and Wnt signaling (Gao et al., 1999; Zou, 2004; Limoni and Niquille, 2021; Yuasa-Kawada et al., 2023); ii) axon projection towards the target area; iii) synapse establishment between target neurons; iv) branching of the axonal tip to form collaterals that innervate multiple neurons; v) synaptogenesis followed by elimination or pruning of extraneous synapses, and strengthening and elaboration of remaining synapses.

Axons originate from axonal growth cones and are influenced by various environmental signals that guide their path through developing neuronal tissue to reach their targets (Yuasa-Kawada et al., 2023). Axon guidance molecules play critical roles in directing the movement of growth cones and the response of neurons to these cues is dependent on expression of specific receptors and intracellular signaling. Molecules that contribute to growth cone guidance include semaphorins, plexin, neuropilin receptors, netrins, Slits and roundabout (Robo) receptors, ephrins and Eph receptors, sonic hedgehog (Shh), bone morphogenetic proteins (BMPs) and Wnt proteins (Yuasa-Kawada et al., 2023). Therefore, characterizing whether developmental guidance molecules could be repurposed to promote correct reconnections of regenerated axons would be an important milestone in the regeneration field.

Axonal development starts with establishment of the anterior-posterior (A-P) axis and dorsal-ventral neuroaxis. A-P axis neurons originate from the dorsal spinal cord commissural neurons that form the ascending somatosensory pathways. These commissural axons grow towards the middle of the floor plate then cross the midline and turn anteriorly toward the brain (Zou et al., 2000). This vertical growth requires attractants netrin-1 and sonic hedgehog along with repellents in the BMP family (Charron et al., 2003). After the axons cross the midline, they are guided by repellents Slit and semaphorin proteins, which transitions axons from a dorsal-ventral axis to an anterior-posterior axis. The role of Slit2 in axon growth across the midline was studied using *Drosophila* and mouse models. Zou et al. demonstrated that after crossing the midline, commissure axons acquire responsiveness to the midline repellent Slit, which induces repulsive cues that prevent axons from re-crossing in the same area (Zou et al., 2000). Wnt signaling facilitates the sharp rostral anterior turn of the axons. Lyuksyutova et al. showed in mice that A-P guidance is also controlled by diffusible gradients of Wnt1, Wnt4, Wnt5a, Wnt6 and Wnt7b ligands that function as attractants to guide axons to extend anteriorly (Lyuksyutova et al., 2003). The researchers demonstrated treatment with gradients of various Wnt molecules caused axons to extend anteriorly through cell aggregates placed next to explanted cultured rat spinal cords (Lyuksyutova et al., 2003). Further investigation confirmed the role of Wnt ligands in developmental axonal growth and guidance by showing that Fzd3 knockout mice had axonal guidance miscues after midline crossing (Lyuksyutova et al., 2003).

Midline axon crossing enables the convergence of axons originating from separate embryonic sides, starting at the telencephalon level. In developing zebrafish embryos, Wnt signaling plays a key role in initiating midline crossing through the Fzd3 receptor. Loss of the Fzd3 prevented glial bridge formation and lead to complete loss of the anterior commissure (Armstrong et al., 2011). Furthermore, lack of Fzd3 signaling caused increased Slit2 expression, resulting in absent or aberrant axonal crossing. These results demonstrate a link between Wnt-induced axonal crossing and Slit-induced repellent cues for correct pathfinding in axons.

Wnt signaling also contributes to guiding axonal crossing during ocular development. Using a mouse model, Wnt5a in the optic chiasm was shown to stimulate retinal axon crossing through an alternative non-canonical Wnt pathway that involves β-catenin accumulation but does not lead to canonical Wnt signaling activation (Morenilla-Palao et al., 2020). Additionally, the transcription factor Zic2 plays a role in facilitating the transition to the alternate Wnt pathway by regulating expression of various Wnt receptors and intracellular proteins, including Sox4, Brn3a, Isl2, Fzd1, Fzd8 and Lgr5. Concurrently, EphB1 activation at the midline induced a repellent response involving phosphorylated β-catenin in the alterative pathway (Morenilla-Palao et al., 2020). Additional studies determined that genetic loss of Apc1, an antagonist of Wnt/β-catenin, in mutant zebrafish resulted in retinal disorganization and loss of axonal crossing and pathfinding within the optic chiasm. Furthermore, axon pathfinding and crossing were corrected when wild type cells from non-mutant zebrafish were transplanted onto the retina of zebrafish expressing mutant Apc1 (Paridaen et al., 2009). These studies indicate Wnt plays an essential role in axonal crossing within the developing optic system.

It is notable that the role of Wnt signaling in axon guidance is not uniform across all neural pathways. For example, non-canonical Wnt signaling has a repulsive effect on corticospinal tract (CST) axons. During neurodevelopment, CST neurons begin pathfinding at the cervical and thoracic levels and are guided by repulsive cues provided by Wnt1 and Wnt5a ligands. The neonatal spinal cord secretes Wnt ligands creating the anterior-posterior gradient that acts as a repellant to corticospinal axons. This gradient provides stronger anterior repulsion and causes CST axons to descend (Liu et al., 2005). Lui et al. showed that a high-to-low gradient of Wnt ligands in the anterior-posterior axis helps initiate the descending guidance of CST axons. Wnt1 and Wnt5 act through the canonical and non-canonical pathway to activate Ryk receptors present in CST axons that leads to repellant signals. Inhibiting the Ryk receptor in mice using antibodies against Ryk prevents axon repulsion by Wnt signaling, indicating the importance of Ryk in Wnt-dependent repulsion of axons towards their intended targets (Liu et al., 2005).

The interaction of Wnt signaling with other developmental signaling pathways during neuronal development was demonstrated for synaptic dendritic branching in mice. Mice transgenic for Wnt7b show activated JNK, which is a kinase pathway that is essential for dendritic outgrowth development, and JNK inhibition led to loss of Wnt-induced developmental dendritic branching (Rosso et al., 2005). Therefore, regulation of multiple neuronal development pathways by Wnt signaling proteins during CNS development suggests potential mechanisms for axonal regeneration after injury.

### Developmental genes promote axonal regeneration after CNS injury in adults

A major research question for neurorepair and axonal regeneration is whether the developmental factors described above can initiate axonal regrowth following injury in adult neural tissue. Axonal regeneration within the CNS is limited due to various molecular and cellular barriers, in contrast to regenerating peripheral nerves. Secretion of inhibitory factors by oligodendrocytes, such as myelin-associated inhibitors, chondroitin sulfate proteoglycan (CSPG), and cytokines creates an inhibitory environment for axon growth within the CNS. In regenerative tissue, such as zebrafish CNS, the surrounding extracellular matrix is modified to allow for axon regrowth in this inhibitory environment. In contrast, mammalian neuronal injuries cause release of molecules at the injury site that convert the extracellular matrix into an inhibitory environment that prevents axons from traversing the injury site, therefore hindering regeneration.

Various amounts of axon regeneration are achievable following injuries to the optic nerve, spinal cord and brainstem. Importantly, recent work showed that neurons revert to an embryonic state in response to such injuries (Zheng and Tuszynski, 2023). A transcriptomics analysis was used to understand regenerative potential within the mouse CNS in which spinal cord injury was followed by neural progenitor cell graft (NPC) transplantation into the injured area. Analysis of translating ribosome affinity demonstrated a “regenerative transcriptome” was induced following spinal cord injury and NPC grafting that included embryonic developmental genes such as SOX2, Wnt ligands and mTOR. An identical transcriptome was initially identified in mice that were injured without receiving an NPC graft, but after 2 weeks, mice that did not receive an NPC graft showed a reduction in the regenerative transcriptome, while mice that did receive the graft maintained the regenerative transcriptome. These intriguing results suggest that cortical spinal neurons are capable of reverting back to an embryologic transcriptional state and provide the basis for exploring the link between embryologic development and regeneration (Poplawski et al., 2020).

The therapeutic applications of manipulating developmental genes to induce repair or regeneration of the CNS are in the early stages of investigation. Several molecular pathways associated with ocular development have been shown to induce axonal regeneration after CNS injury, suggesting possible overlap of signaling pathways between development and regeneration after nerve damage (Figure 2). The following signaling pathways and factors have been implicated in development and axonal repair after CNS injury.

### Wnt signaling in axonal regeneration

Wnt pathway components may overcome inhibitory conditions following CNS injury and promote axonal regeneration (Akram et al., 2022). Wehner et al. demonstrated that Wnt/β-catenin signaling is upregulated in non-neural fibroblast-like cells at the lesion site after spinal cord transection in zebrafish. Activation of Wnt/β-catenin signaling using Wnt8 induced expression of the alpha chain of collagen XII, which allowed axons to navigate across non-neural cells within the injury site and resulted in regeneration. Inhibiting Wnt/β-catenin signaling caused downregulation of the gene encoding collagen XII, *col12a1a/b*, and decreased regeneration. In contrast, simultaneous overexpression of *col12a1a/b* and inhibition of Wnt/β-catenin signaling resulted in complete rescue of axonal bridging to wild-type levels, indicating the ability of collagen XII to counteract inhibition of axonal regrowth (Wehner et al., 2017). Furthermore, overexpression of the Wnt/β-catenin inhibitor Dkk1b downregulated Wnt/β-catenin signaling and blocked spinal cord regeneration in the zebrafish model. Dkk1b overexpression inhibited glial bridge formation, axonal regeneration, and locomotor recovery of zebrafish (Strand et al., 2016). Therefore, these studies demonstrate that Wnt signaling promotes axonal regeneration following spinal cord injury in zebrafish and mammals.

A recent study by Musada et al., 2020 showed upregulation of endogenous non-canonical Wnt5a ligand after optic nerve injury and delivery of recombinant Wnt5a promoted axonal regrowth and retinal ganglion cell (RGC) survival. The axon regenerative and neuronal survival function of Wnt5a was associated with increased JNK and CamKII activation, implicating specific signaling pathways and their downstream targets in Wnt-mediated axonal regrowth (Musada et al., 2022). Additionally, the canonical pathway ligand Wnt3a showed axonal growth promoting effects in the injured optic nerve. Intravitreal injection of Wnt3a in a mouse model of optic crush injury resulted in increased RGC survival and axon regeneration that was dependent on Stat3 signaling (Patel et al., 2017). Müller glia and RGCs were the target cells for Wnt3a, suggesting that Müller glia contribute to Wnt3a-mediated axonal regeneration (Musada et al., 2020). Similarly, another study using spinal cord injury demonstrated that Wnt3a induced neurite outgrowth and induction of neural stem cells into neurons at the injury site (Yin et al., 2008). Canonical Wnt signaling induces transcription of numerous genes, including the transcription factor c-myc, which is associated with axonal regeneration in adult nervous tissue. Proteomic analysis of FACS-sorted mouse RGCs demonstrated decreased c-myc expression in RGCs following optic nerve injury whereas overexpression of c-myc enhanced RGC survival and promoted axonal regrowth after optic nerve injury (Belin et al., 2015).

The relationship between canonical signaling stimulated by Wnt10b and the mTOR pathway in axonal regeneration was also examined. Fibroblast-derived exosomes containing Wnt10b led to mTOR activation and resulted in increased axonal regeneration after optic nerve injury (Tassew et al., 2017), demonstrating another Wnt ligand that promotes axonal regeneration in adult CNS.

Although Wnt ligands Wnt3a, Wnt5a and Wnt10b increase axonal growth after injury in the CNS (Yin et al., 2008; Patel et al., 2017; Tassew et al., 2017; Musada et al., 2022), other studies showed that Wnt1 and Wnt5a ligands stimulated the non-canonical Wnt-Ryk pathway and inhibited axonal growth in spinal cord injuries (Miyashita et al., 2009). Treatment of cerebellar granule neurons (CGNs) from young rat pups with Wnt5a resulted in Wnt-Ryk signaling, activation of RhoA/Rho-kinase and decreased neurite growth (Miyashita et al., 2009). Wnt-Ryk signaling induced axon repulsion cues, leading to corticospinal axon retraction and branching after injury (Liu et al., 2008). Furthermore, blocking Ryk after spinal cord injury led to neurite outgrowth. A study by Miyashita et al. demonstrated that Wnt-Ryk signaling inhibited axonal regrowth after damage. Overexpression of Wnt5a in astrocytes around the thoracic spinal cord contusion injury site in a rat model led to axonal outgrowth inhibition. In contrast, administration of an anti-Ryk antibody rescued corticospinal neurite growth and functional recovery in adult rats following thoracic spinal cord contusion. Additionally, Hollis and Zhou demonstrated an inhibitory role for Wnt-Ryk in axonal regeneration. Their study revealed increased Ryk expression following injury to the central branches of dorsal root ganglion neurons and inhibiting Wnt-Ryk signaling using bone marrow stromal cells expressing WIF1 and SFRP2 led to axonal regrowth of ascending sensory axons (Hollis and Zou, 2012). However, it is possible that axonal growth inhibition by these Wnt ligands is due to Wnt inducing axonal guidance and pathfinding cues, which cause axons to slow or stop growing in a specific direction. Together, these studies demonstrate the varied beneficial and inhibitory roles of Wnt signaling in axonal regeneration (Figure 2).

### BMP signaling

BMP signaling proteins belong to a protein family that contributes to mammalian eye development, including lens formation and retinal development. Wnt and BMP signaling pathways intersect and coordinate various processes during development. For example, Wnt signaling regulates BMP function during retinal development in zebrafish, as shown in a study by Veien et al. Inhibiting Wnt signaling using Dkk1 lead to reduced BMP4 expression and increased expression of ventral genes such as Vax2 in dorsal areas, indicating that suppressing Wnt signaling caused increased ventralization (Veien et al., 2008). These findings demonstrate that BMP-Wnt crosstalk is necessary for dorsal retinal gene expression. Furthermore, delivering BMP rescued expression of the dorsal gene Tbx5 in embryos expressing Dkk1, but blocking BMP signaling and reactivation of Wnt signaling was unable to rescue Tbx5 expression. Therefore, Wnt signaling maintains dorsal retinal genes through the regulation of BMP, and specific Wnt-BMP interactions are important for proper eye development (Veien et al., 2008). Additionally, retinal dorsal patterning in mice is influenced by BMP expression levels. Although loss of Bmpr1b resulted in normal eye development, combined loss of Bmpr1b and Bmpr1a caused altered retinal dorsoventral patterning. Additionally, compound mutations in Bmpr1b and 1a resulted in severe eye developmental malformations, including failure of retinal neurogenesis and reduced retinal growth (Murali et al., 2005).

The role of various BMPs in axonal growth and neurite development following injury has also been demonstrated. Inhibiting BMP signaling using the BMP inhibitor Noggin in a mouse PNS injury model using sciatic nerve crush resulted in reduced neurite outgrowth (Ma et al., 2011). BMPs were also shown to promote neurite outgrowth in cultured RGCs. In a study using cultured postnatal rat retinas, BMP2 and BMP13 enhanced overall neurite number, neurite length and number of neurite branch points (Kerrison et al., 2005). Another study showed that BMP4/Smad1 signaling promoted axonal regeneration in the spinal dorsal column. Endogenous BMP4 and Smad1 expression levels were elevated in the dorsal column following crush injury in mice. BMP4 over-expression using AAV8 in rats induced axonal regeneration and rescued dorsal column function as measured by a horizontal ladder crossing test (Farrukh et al., 2019). BMP molecules were also recently implicated in inhibiting differentiating oligodendrocyte progenitor cells (OPC) following demyelination after CNS injury. Inhibition of OPC differentiation involved interactions of Sulf2, an extracellular endosulfatase, with BMP and Wnt signaling. Sulf2 acts through BMP signaling to create an inhibitory environment that reduced OPC myelination (Saraswat et al., 2021). These studies demonstrate the potential of BMPs as potential therapeutic targets for promoting axonal regeneration in the CNS.

### Krüppel-like factors (Klf)

Several transcription factor families play significant roles in CNS development and are potential drivers of axonal regeneration pathways. For example, Krüppel-like factors (Klf) are involved in embryologic neurogenesis and play roles in RGC axonal growth in culture (Laub et al., 2001; Veldman et al., 2007). Various Krüppel subtypes display opposite functions *in vitro*. For example, overexpression of Klf-4 and -9 in RGC cultures inhibited neurite growth whereas Klf-6 and -7 promoted neurite growth of RGCs *in vitro* (Moore et al., 2009) and induced spinal cord axon growth in adult mice (Blackmore et al., 2012). Additionally, genetic deletion of Klf4 resulted in axon growth following optic nerve injury in mice, providing another example of a developmental gene that regulates axonal regeneration in adults (Moore et al., 2009). Klf4 inhibits Wnt signaling by binding to β-catenin in the intestinal epithelium (Evans et al., 2010), but whether Wnt and Klf4 interact during CNS axonal regeneration after injury is not known.

### Pax6

Another embryologic gene linked extensively to eye development is the transcription factor PAX6, which acts during early embryogenesis to initiate retinal development. Pax6 plays a crucial role in the development of the optic vesicle and its absence leads to complete loss of the optic vesicle. Pax6 is expressed throughout the surface ectoderm and neuroepithelium and plays roles in the differentiation of the cornea, lens, ciliary body, retina, and iris (Klimova and Kozmik, 2014). Pax6 also influences post-natal eye development and is essential for differentiation of glycinergic amacrine cells, bipolar cells and photoreceptors (Remez Liv et al., 2017). Pax6 and Wnt pathways interact during CNS development. For example, Pax6 mutant mice show reduced Wnt7b expression in developing spinal cord (Kim et al., 2001) and Pax6 inhibits canonical Wnt signaling by regulating expression of several Wnt inhibitors in the developing lens (Machon et al., 2010). Despite its prominent role in ocular development, investigation of the role of Pax6 in axonal regeneration is limited. An early study using Pax6 transfection into differentiating mouse neural stem cell-derived neurospheres demonstrated that cells overexpressing Pax6 had increased axonal extensions compared to controls, suggesting a potential role of Pax6 in stimulating axonal growth (Sebastián-Serrano et al., 2012). Interactions between Wnt and Pax6 during axonal regeneration after injury have not been reported. Further research is needed to understand the axonal regenerative capacity of Pax6 before it can be considered as a candidate regulator of axonal growth after injury.

### Sox

Sox is a transcription factor gene family associated with eye development. Sox2 and Wnt signaling have complex interactions during retinal development and Sox2 inhibits Wnt signaling during neuronal differentiation (Agathocleous et al., 2009), although it is unknown whether Wnt and Sox2 interact during axonal regeneration after injury. Sox gene family members play various roles in development and are expressed in neural progenitors and stem cells in the retina and throughout the CNS. For example, Sox2 expression is maintained during optic disc formation with restriction to neural retinal cells whereas Sox1 and Sox3 are downregulated during optic disc formation. Sustained expression of Sox2 suggests it has a significant role in retinal development (Taranova et al., 2006). Sox2 expression within postnatal neural progenitor cells in mice results in differentiation to Müller glia. Forced over expression of Sox2 without retinal damage resulted in Müller glia proliferation, indicating the role of Sox2 in re-entry into the cell cycle (Surzenko et al., 2013). Although Sox2 has not been shown to directly promote axonal regeneration, the involvement of Sox2 in Müller glia-derived neuron replacement indirectly implicates Sox2 in potentially forming new axons from regenerated neurons in response to retinal damage.

### Ephrins and p75^NTR^

Ephrins are a large ligand-receptor family involved in eye development that regulates axonal guidance by functioning as axonal repellants. Ephrins inhibit Wnt signaling in developing retina (Fang et al., 2013) but the interactions of the pathways during axonal regeneration after injury have not been investigated. Previous studies demonstrated that multiple ephrins were upregulated following CNS injury (Willson et al., 2003; Duffy et al., 2012). A study by Ren et al. identified the role of ephrin-B2 in inhibiting axonal regeneration after spinal cord injury in mice. Astrocyte-specific ephrin-B2 knockdown mice demonstrated enhanced corticospinal axonal regeneration as well as decreased astrogliosis and scaring, which highlights the significance of astrocyte-derived ephrin-B2 in impeding axonal regeneration post-injury (Ren et al., 2013) and links inhibitory ephrin-B2 function to excessive astrocyte activation. Chen et al. demonstrated using EphA4 knockout mice that astrocyte cytokine secretion is altered by EphA4 and ephrin-B ligands. Furthermore, EphA4 promotes secretion of adhesin and certain cytokines, inhibits IL-1β and IL-6 release and promotes glial scar formation. RNA interference studies demonstrated that EphA4 inhibited neurite outgrowth when bound to ephrin-B1, ephrin-B2 and -B3, and reducing the expression of these molecules increased neurite regeneration (Chen et al., 2022). These findings indicate that EphrinA4 and ephrin-B1 on astrocytes are potential therapeutic targets for neurite regeneration. Additionally, in a recent study performed on post-natal mouse retinas after optic nerve crush, inhibition of EphA and EphB resulted in a significant protective effect on RGC dendritic arborization (Strong et al., 2023). These results provide further support for axonal regenerative regulating activity of ephrins and identify possible treatment strategies for further investigation.

The p75 neurotrophin receptor (p75^NTR^) binds to neurotrophins and non-neurotrophin ligands and is essential for synapse stabilization and neuronal maturation. p75^NTR^ is implicated in regulating axonal growth by binding to inhibitory myelin proteins such as Nogo, MAG and MOG, which interact in a p75^NTR^/Nogo receptor complex (Meeker and Williams, 2015). p75^NTR^ also mediates the effects of ephrins. During axonal elongation, p75^NTR^ allows RhoA to interact with Rho-GDP dissociation inhibitor, preventing activation of Rho guanine nucleotide exchange factors. Neurons cultured with ephrin B3 and a Rho inhibitor showed decreased neurite outgrowth, indicating ephrin B3 inhibits growth through Rho. Furthermore, the inhibitory effect of ephrinB3 on neurite growth was lost in mice with p75^NTR^ knock down, indicating p75^NTR^ is required for axonal growth inhibition. Treatment with Pep5, an inhibitor of p75^NTR^ and Rho-GDP interaction, reversed the inhibitory effects of ephrin B3 on axonal growth. Additionally, suppression of ephrin B3 by blocking the p75^NTR^ complex leads to axonal regeneration after optic nerve crush injury in mice (Uesugi et al., 2013). These findings demonstrate that ephrin B3 acts through p75^NTR^–RhoA signaling. Therefore, ephrins are important repellant molecules that when inhibited promote axonal regeneration in the optic nerve.

### JAK/STAT pathway

Ciliary neurotrophic factor (CNTF), which stimulates JAK/STAT signaling, and the inhibitor suppressor of cytokine signaling 3 (SOCS3), have also been shown to regulate axonal growth during development as well as axonal regeneration after injury in mouse models. CNTF is a neurotrophic factor implicated in regulating astrocyte formation from spinal cord derived progenitor cells (Nakamura et al., 2011). CNTF has also been shown in multiple studies to promote axonal regeneration in injured adult neuronal tissue (Sendtner et al., 1991), although CNTF did not provide sufficient axonal guidance cues (Pernet et al., 2013). A study performed by Müller et al. demonstrated CNTF enhanced axonal growth through the JAK/STAT3 pathway (Müller et al., 2009). Further investigation into the role of the JAK/STAT pathway demonstrated reduced axonal growth in the presence of SOCS3, which acts as a negative regulator of STAT signaling (Smith et al., 2009). Combining SOCS3 and CNTF reduced axonal regeneration within the mouse optic nerve, whereas SOCS3 knockdown in RGCs promoted axonal regeneration (Smith et al., 2009). Recent work also demonstrated that Wnt5a functions through JAK/STAT3 activation to increase axonal growth following optic nerve injury (Musada et al., 2022). These results implicate the JAK/STAT3 pathway as an important regulator of axonal regeneration and growth.

### PTEN

The phosphatase PTEN inhibits RGC axonal growth in adult mice following optic nerve injury through negative regulation of the mTOR pathway. PTEN knockout mice showed increased axonal regeneration after optic nerve damage (Park et al., 2008) and loss of PTEN in human embryonic stem cell derived neuronal progenitors resulted in increased neurite outgrowth (Wyatt et al., 2014). PTEN cross-talks with other signaling pathways, including Wnt. For example, PTEN controls secretion of Wnt ligands during retinal differentiation (Touahri et al., 2024) but whether PTEN regulates Wnt-dependent axonal regeneration is unknown.

### The role of Müller glia in neuronal regeneration

Müller glia have recently been shown to play key roles in cellular regeneration in various animal models of retinal injury. Müller glia are radial glia in the retina that have the ability to be reprogrammed from their quiescent state into a highly proliferative state following injury. This phenomenon has been demonstrated in zebrafish and mouse models. Several transcription factors were identified based on comparison of their expression patterns between mouse Müller glia and retinal progenitors and were investigated for their ability to initiate neurogenesis in Müller glia *in vitro* (Pollak et al., 2013). Among the factors tested, the proneural basic helix-loop-helix (bHLH) transcription factor Ascl1 was able to stimulate neurogenesis from Müller glia in young and adult mouse retinas when co-administered with the histone deacetylase inhibitor trichostatin A (Jorstad et al., 2017). The expression of Ascl1 in Müller glia led to the acquisition of a molecular phenotype resembling that of developing retinal progenitors, with a subset of these cells undergoing mitotic division (Jorstad et al., 2017; Todd et al., 2020). Notably, some of the newly generated cells differentiated into retinal neurons that generated synaptic connections with existing neurons (Jorstad et al., 2017). These studies highlighted the potential for Müller glia as a source of neuronal replacement following injury, which could potentially lead to axonal regeneration following completed differentiation of the new neurons and integration within the CNS. However, the mechanisms by which newly formed neurons could be instructed to successfully incorporate their regenerated axons into the existing CNS circuitry remains unclear, and regrowing axons from pre-existing neurons is likely a more efficient strategy.

The role of the inflammatory transcription factor Nfkb1 in Müller glia-mediated neuron regeneration has been investigated in mouse retina. Palazzo et al. found that Nfkb1 acts as a coordinator of Müller glia reprogramming (Palazzo et al., 2022) and mice with Müller glia-specific Nfkb1 knockdown had more glial-derived neurons. Therefore, Nfkb1 inhibition combines with Ascl1 to stimulate Müller glia reprogramming and induce neuronal regeneration (Palazzo et al., 2022).

Another interesting pathway linked to Müller glia reprogramming and developmental embryology is the Hippo-YAP/TAZ pathway. The Hippo pathway is highly conserved among species and is activated in response to changes in cellular structure and nutrient availability, thus acting as a sensor of cellular health and stability. Upon Hippo activation, its co-activators YAP/TAZ interact with TEAD transcription factors (TEAD1–TEAD4) forming a complex that associates with gene enhancers. These complexes interact with chromatin-remodeling factors and regulate RNA polymerase II activity to either promote or inhibit target gene expression, including various regulators of the cell cycle, cell migration, and cell fate (Moya and Halder, 2019).

YAP/TAZ signaling has been associated with axial formation and organogenesis. Indeed, loss of YAP1 in mice led to significant abnormalities in body axis deformation, resulting in shortened body axis length and defects in neuroepithelium formation and yolk sac vascularization (Moya and Halder, 2019). Further research has implicated Hippo-YAP/TAZ in Müller glia-derived neuronal regeneration. YAP1 knockdown in zebrafish prevented Müller glia transitioning to a reactive proliferative state and abolished regeneration (Hoang et al., 2020). Additionally, overactivated YAP1 in mouse Müller glia was sufficient to convert previously quiescent Müller glia into highly proliferative cells following retinal injury, although no new neurons were formed (Hamon et al., 2019). The results of these studies provide valuable insights in understanding a neuronal regenerative pathway and also identify key molecules that could promote therapeutic cell regeneration.

### New approaches for identifying mediators of axonal regeneration

Exciting new technologies are rapidly increasing identification and understanding of axonal regeneration mediators. One of the technologies commonly utilized is single cell transcriptomics such as single cell RNA-sequencing (scRNAseq), which allows precise quantification of the transcriptomes of individual cells. A recent report used scRNAseq to profile gene expression of ten developmental stages encompassing the full course of retinal neurogenesis in mice (Clark et al., 2019), which provides an important resource for identifying regulatory genes and their targets. Another emerging technology similar to scRNAseq is spatial transcriptomics which allows mapping of the whole transcriptome while maintaining spatial context. This method allows examination of adjacent cells at a molecular and genomic scale previously not available, which opens avenues for further research into specific genes that could be explored for regenerative purposes (Rao et al., 2021; Williams et al., 2022). Spatial genomics has recently been applied in a study identifying the spatial resolution changes observed in mice retinas during development (Dorgau et al., 2024). Additionally, advances in multi-omic analyses have been used to compare growth cone composition during axonal growth in embryonic and adult CNS. These analyses identified changes in protein and lipid mediators during growth cone extension and provided new understanding of lipid metabolism in growth cone dynamics during axonal regeneration (Chauhan et al., 2020).

There are many essential questions in the field that require additional investigation using new and existing technologies. For instance, it is crucial to determine how various developmental signals, which are suppressed after neuronal development is completed, are reactivated following an injury and how these signals interact to coordinate axonal regeneration. Additionally, it is important to recognize how differences between injured and developing CNS tissue affect neuronal responses to proregenerative stimuli. It is also essential to consider potential off-target effects of therapeutic interventions, as certain factors like BPMs and Wnt have been linked to angiogenesis and tumorigenesis and the timing and expression levels of these pathways will influence the extent of adverse effects. Therefore, future therapeutic application would need to incorporate technologies that enable precise cell targeting and axonal guidance, while also allowing for the termination of gene expression after newly regrown axons have established connections with their intended synaptic partners.

## Conclusion

As discussed above, proper development relies on the coordination of multiple molecular pathways. Of particular significance are the Wnt signaling pathways, which play crucial roles in regulating various aspects of neural development and were demonstrated to induce neuronal survival and axonal regeneration in adult CNS in various animal injury models. Experimental evidence suggests other genes implicated in key developmental processes also exhibit axonal growth-regulating effects after injury, including Ephrins, KLF4, BMPs, CNTF/Stat3, Notch and Slit. Moreover, exploration of regulatory genes and pathways such as Wnt, BMP, Pax, and Sox offers promising avenues for promoting therapeutic axonal regrowth and regeneration. The manipulation of Wnt and other pathways holds great potential for understanding and advancing the field of axonal regeneration. In conclusion, there are multiple areas of molecular overlap between developmental embryology and neuronal repair and regeneration, and substantial research efforts to date have set the foundation for future therapeutic applications.

### Scope statement

A major research focus in the Neuroscience field is identifying axon regenerative mechanisms in adult CNS after injury. Interestingly, several embryonic axon growth factors have been repurposed as axon regenerative promoting factors in adult CNS in various animal injury models. In this review article, we examine the existing literature on the extent of overlap of embryonic axon growth and guidance activities and axon regenerating functions. We describe evidence for potential contributions of various embryonic genes and transcription factors involved in eye development for their potential roles in axonal regeneration, and highlight major research gaps. We also review emerging technologies that have been used to identify candidate axonal growth molecules in developing and adult tissues. Therefore, this manuscript provides a new summary of the field and highlights the advantages of integrating embryonic developmental biology into neural axonal regeneration studies. This manuscript aligns with the multidisciplinary focus of the Frontiers journal by combining different fields of study to illustrate their interconnectedness.

## Figures and Tables

**Table:** 

**bHLH**	Proneural basic helix-loop-helix
**BMP**	Bone morphogenetic protein
**Bmpr**	Bone morphogenetic protein
**CaMKII**	Ca^2+^/calmodulin-dependent protein kinase II
**CGN**	cerebellar granule neurons
**CNS**	Central Nervous System
**CNTF**	Ciliary neurotrophic factor
**CREB**	cAMP-response element binding
**CSPG**	Chondroitin sulfate proteoglycan
**CST**	Corticospinal tract
**Dkk**	Dickkopf
**Dvl**	Dishevelled
**Eph**	Ephrin
**FZD**	Frizzled
**GSK3β**	Glycogen Synthase Kinase 3β
**JNK**	c-Jun N-terminal kinase
**Klf**	Krüppel-like factor
**LRP**	Low-density lipoprotein receptor
**MAG**	Myelin-associated glycoprotein
**MOG**	Myelin oligodendrocyte glycoprotein
**mTOR**	Mammalian target of rapamycin
**NFAT**	Nuclear factor of activated T cells
**Nfkb1**	Nuclear factor kappa-beta
**Notch**	Neurogenic locus notch homolog
**NPC**	Neural progenitor cell graft
**p75^NTR^**	p75 neurotrophin receptor
**OPC**	oligodendrocyte progenitor cells
**PCP**	Wnt/planar cell polarity
**PKC**	Protein kinase C
**PLC**	Phospholipase C
**PTEN**	Phosphatase and tensin homolog
**Rac**	Rac family small GTPase
**RGC**	Retinal ganglion cell
**Rho**	Ras homolog gene family GTPase
**ROCK**	Rho kinase
**ROR1/2**	Receptor tyrosine kinase-like orphan receptor 1/2
**Rspo**	R-spondin
**Ryk**	Receptor like tyrosine kinase
**scRNAseq**	Single cell RNA-sequencing
**sFRP**	Secreted frizzled receptor proteins
**Smad**	Suppressor of mothers against decapentaplegic
**SOCS3**	Suppressor of cytokine signaling 3
**Sox**	SRY-box
**TCF/LEF**	T-cell factor/lymphoid enhancer
**TFGB**	Transforming growth factor-beta
**WIF**	Wnt Inhibitor Factor
**Wnt**	Wingless-related integration site
